# Mutant Cu/Zn Superoxide Dismutase (A4V) Turnover Is Altered in Cells Containing Inclusions

**DOI:** 10.3389/fnmol.2021.771911

**Published:** 2021-11-03

**Authors:** Natalie E. Farrawell, Justin J. Yerbury

**Affiliations:** ^1^Illawarra Health and Medical Research Institute, Wollongong, NSW, Australia; ^2^School of Chemistry and Molecular Bioscience and Molecular Horizons, University of Wollongong, Wollongong, NSW, Australia

**Keywords:** ALS, SOD1, UPR – unfolded protein response, UPS – ubiquitin proteasome system, proteome homeostasis

## Abstract

SOD1 mutations account for ∼20% of familial amyotrophic lateral sclerosis (ALS) cases in which the hallmark pathological feature is insoluble SOD1 aggregates within motor neurons. Here, we investigated the degradation and synthesis of mutant SOD1 to determine whether the aggregation of mutant SOD1^A4V^ affects these processes. We confirm that, in general, the degradation of mutant SOD1^A4V^ occurs at a significantly faster rate than wild-type SOD1. We also report that the turnover and synthesis of mutant SOD1^A4V^ is impaired in the presence of insoluble SOD1^A4V^ aggregates. However, the timing of aggregation of SOD1^A4V^ did not coincide with UPS dysfunction. Together, these results reveal the impact of SOD1 aggregation on protein degradation pathways, highlighting the importance of the UPS in preventing neurodegenerative disorders such as ALS.

## Introduction

Amyotrophic lateral sclerosis (ALS) is a fatal neurodegenerative disease characterised by the progressive loss of motor neurons in the brain and spinal cord. Although most cases of ALS are sporadic with no known cause (sporadic ALS; sALS), approximately 10% of cases have a known family history of the disease (familial ALS; fALS). Dominant missense mutations in the gene encoding superoxide dismutase 1 (SOD1) were the first discovered to cause ALS, and account for approximately 20% of all fALS cases and up to 50% in China. However, there are now a plethora of genes associated with ALS. Strikingly, a large proportion of these genes including *VCP*, *SQSTM1*, *UBQLN2*, *OPTN*, *TBK1*, *CCNF*, *DNAJC7* and *CYLD*, are associated with protein degradation pathways, one of the three main functional pathways proposed to perturb proteome homeostasis (Yerbury et al., 2020).

Consistent with a collapse in proteome homeostasis, the pathology of ALS is characterised by the deposition of ubiquitinated protein inclusions within motor neurons (Leigh et al., 1991). The composition of these inclusions is heterogeneous, and the primary constituent varies depending on the disease subtype (sALS or fALS), and the underlying gene mutation. While SOD1 fALS cases show exclusive deposition of SOD1, inclusions also contain proteins associated with quality control machinery and other unrelated aggregation-prone or supersaturated proteins (Ciryam et al., 2013, 2015). These supersaturated proteins are over-represented in biochemical pathways known to be disrupted in ALS, including protein production, protein trafficking and protein degradation (Ciryam et al., 2013, 2017). It is still widely debated whether protein aggregation is a cause or consequence of disease, but numerous studies have implicated a correlation between aggregate load and neurotoxicity in ALS (Leigh et al., 1991; Matsumoto et al., 2005; Ticozzi et al., 2010; Brettschneider et al., 2014). Misfolded and aggregated SOD1 elicit many toxic properties; impaired axonal transport (Bosco et al., 2010), activation of endoplasmic reticulum (ER) stress (Nishitoh et al., 2008), and self-propagation in a prion-like fashion (Strong et al., 2005; Munch et al., 2011; Grad et al., 2014; Zeineddine et al., 2015). Moreover, our work in NSC-34 cells identified a correlation between aggregation propensity and rate of disease progression (McAlary et al., 2013), suggesting protein aggregation is linked with cell death.

Previous studies have shown that mutant SOD1 proteins are turned over more rapidly than wild-type SOD1 (Borchelt et al., 1994; Hoffman et al., 1996; Johnston et al., 2000; Miyazaki et al., 2004), and SOD1 proteins are degraded by both the ubiquitin proteasome system (UPS) and autophagy pathway (Kabuta et al., 2006). However, very little research has been undertaken to assess the impact of SOD1 aggregation on SOD1 protein turnover. A previous study examined the rate of SOD1 turnover in mouse spinal cord and found no difference regardless of the amount of aggregation and level of disease progression (Farr et al., 2011). Although, the rate of turnover in individual cells containing inclusions of aggregates remains to be examined. In this study, we confirm that the turnover of mutant SOD1 in NSC-34 cells is considerably faster than that of wild-type SOD1. We also show that SOD1 turnover and synthesis is impaired in cells containing insoluble SOD1^A4V^ aggregates and that UPS dysfunction most likely occurs prior to the aggregation of ALS-associated proteins.

## Materials and Methods

### Plasmid Constructs

SOD1-Dendra2 constructs were generated by cloning human SOD1^WT^ and SOD1^A4V^ into the pDendra2-N vector (Clontech, United States). pEGFP-N1 containing human SOD1^A4V^ was generated as described previously (Turner et al., 2005). The mcherry^CL1^ construct was obtained by cloning the CL1 sequence (ACKNWFSSLSHFVIHL) into pmCherry-C1.

### Cell Culture and Transfection

Neuroblastoma × spinal cord hybrid NSC-34 cells (Cashman et al., 1992) were cultured in Dulbecco’s Modified Eagle’s Medium/Ham’s Nutrient Mixture F12 (DMEM/F12) supplemented with 10% fetal bovine serum (FBS, Gibco, Australia). Cells were maintained at 37°C in a humidified incubator with 5% atmospheric CO_2_. Cells were plated onto 8-well μslides (Ibidi, Germany) 24 h prior to transfection and transfected with 200 ng of plasmid DNA (per well) using Lipofectamine 3000 (Invitrogen) or TransIT-X2 transfection reagent (Mirus Bio, United States) according to manufacturer’s instructions. For co-transfections, the amount of DNA was divided equally between constructs. Immediately prior to imaging, the media on transfected NSC-34 cells was removed and replaced with a FluoroBrite DMEM medium (Gibco) containing 5% FBS.

### Live Cell Imaging

Time-lapse imaging of transfected NSC-34 cells was performed 24 h post-transfection using a Leica DMi8 Thunder fluorescence microscope equipped with an enclosed environmental control chamber, which was maintained at 37°C with 5% CO_2_. For NSC-34 cells expressing SOD1-Dendra2, SOD1-Dendra2-GFP was irreversibly converted to SOD1-Dendra2-RFP by exposure to DAPI LED for 30–60 s at 40% power, using the 20 × objective. Following photoconversion, 3 × 3 tile scans were acquired in each well every 15 min for up to 68 h using continuous adaptive focus control and the 20 × objective in the bright field, GFP (30 ms exposure, 40% power) and Texas Red/RFP (45 ms exposure, 40% power) channels.

For NSC-34 cells co-expressing SOD1^A4V^-GFP and mcherry^CL1^, 3 × 3 images were acquired every 20 min for up to 70 h using the 20 × objective and continuous adaptive focus control in the bright field, GFP (35 ms exposure, 40% power) and Texas Red/RFP (45 ms exposure, 40% power) channels.

### Quantification and Tracking of Cu/Zn Superoxide Dismutase-Dendra2 Fluorescence

A CellProfiler pipeline was developed to quantify the SOD1-Dendra2-GFP and SOD1-Dendra2-RFP fluorescence in photoconverted cells from images produced from the DMi8 Thunder microscope. Firstly, RFP images were rescaled to stretch each image and use the full intensity range. Primary objects (Dendra2-RFP expressing cells) were identified based on size (20–60 pixel units) using an adaptive Otsu (three classes) thresholding method. The RFP fluorescence intensity of identified cells was then measured and exported to a spreadsheet. To compare the degradation of SOD1^WT^-Dendra2 and SOD1^A4V^-Dendra2 over time, the Dendra2-RFP fluorescence intensity of cells within each image was summed at each time point and normalised to time zero.

To track individual cells expressing soluble and insoluble SOD1^A4V^-Dendra2, a separate CellProfiler pipeline was generated where RFP images were smoothed using a Gaussian filter before Dendra2-RFP expressing cells were identified based on size (10–60 pixel units) using a minimum cross entropy thresholding strategy. Individual cells were then tracked using the follow neighbours tracking method. Images of tracked cells, along with the GFP and RFP overlay images, were exported and saved so that cells could be visually inspected for the presence of insoluble SOD1^A4V^ inclusions. The GFP and RFP fluorescence intensity of identified cells was also measured and exported to a spreadsheet. In order to assess the degradation and synthesis of SOD1^A4V^-Dendra2 in each cell, the Dendra2 fluorescence at each time point was normalised to time zero. Dendra2-GFP fluorescence was subsequently normalised against Dendra2-RFP fluorescence to assess synthesis rates.

### Tracking Cells Co-expressing Cu/Zn Superoxide Dismutase^A4V^-GFP and mcherry^CL1^

Images of NSC-34 cells co-transfected with SOD1^A4V^-GFP and mcherry^CL1^ were analysed using a Cell Profiler pipeline that rescaled RFP images to identify primary objects (RFP cells) based on size (10–60 pixel units) and adaptive Otsu thresholding. The GFP intensity of RFP cells was then measured and filtered to identify co-transfected cells. Co-transfected cells were tracked using the follow neighbours method and images were exported as described above to identify cells containing insoluble SOD1^A4V^ inclusions. The RFP and GFP intensities of co-transfected cells were measured and exported to a spreadsheet for analysis. Fluorescence intensity at each time point was normalised to time zero.

### Statistics

All statistical analysis was performed using GraphPad Prism software version 9.00 for Windows unless stated.

## Results

### The Half-Life of Cu/Zn Superoxide Dismutase Is Diminished by Amyotrophic Lateral Sclerosis-Associated Mutations

**FIGURE 1:**
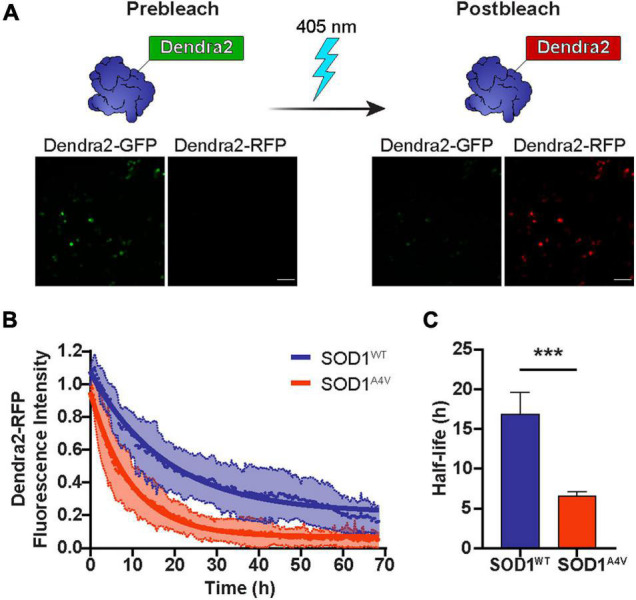
The half-life of SOD1 is diminished by ALS-associated mutations. **(A)** Dendra2-GFP signal in NSC-34 cells was irreversibly converted to Dendra2-RFP by exposure to 405 nm light. Scale bars: 100 μm. **(B)** The degradation of the SOD1-Dendra2-RFP signal in NSC-34 cells was measured over 68 h. Data shown are mean ± SD (*n* = 17) with the solid lines representing non-linear regression curves. **(C)** The half-life of SOD1^WT^-Dendra2 and SOD1^A4V^-Dendra2 was calculated from the degradation of the SOD1-Dendra2-RFP signal in NSC-34 cells. Data represent mean ± SEM of cells acquired from *n* = 17 images and statistical significance between groups was determined using an unpaired Student’s *t*-test (***p < 0.001). Data is representative of at least two independent experiments.

Numerous studies have shown that the turnover of mutant SOD1 occurs at a much faster rate than that of wild-type SOD1 (Borchelt et al., 1994; Hoffman et al., 1996; Johnston et al., 2000; Miyazaki et al., 2004). Here, we monitored the real-time degradation of SOD1 in NSC-34 cells by tagging SOD1 variants with the photoconvertible fluorescent protein Dendra2. Denda2-GFP signal was irreversibly converted to Dendra2-RFP by exposure to 405 nm light (Figure 1A), creating a pool of protein that would remain unchanged unless it was degraded. The photoconverted RFP signal that degraded over time was used as a measure of protein turnover. Consistent with previous reports, we found that the turnover of mutant SOD1^A4V^-Dendra2 was faster in NSC-34 cells than SOD1^WT^-Dendra2 (Figure 1B), with SOD1^A4V^-Dendra2 exhibiting a significantly shorter half-life (∼7 h) compared to SOD1^WT^-Dendra2 (∼17 h) (Figure 1C).

### The Degradation and Synthesis of Cu/Zn Superoxide Dismutase^A4V^ Is Slowed in Cells Containing Insoluble Aggregates

**FIGURE 2:**
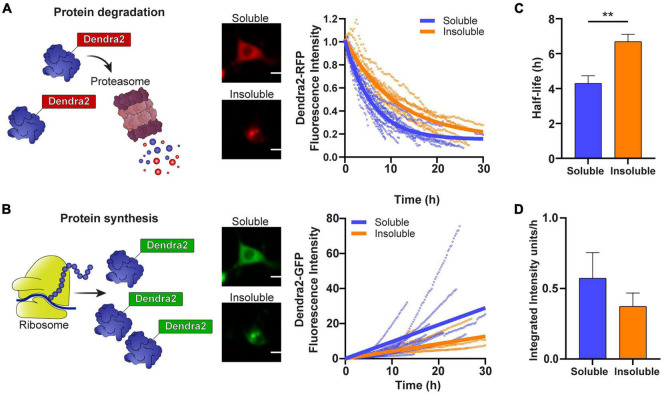
The degradation and synthesis of SOD1 are slowed in cells containing insoluble aggregates. **(A)** The degradation of SOD1-Dendra2-RFP signal and **(B)** synthesis of SOD1-Dendra2-GFP signal was tracked in NSC-34 cells expressing soluble or insoluble SOD1^A4V^ over 30 h. Data shown are representative of non-linear and linear regression curves (solid lines) and individual cell traces (*n* = 18 soluble SOD1^A4V^, *n* = 12 insoluble SOD1^A4V^) from one replicate. **(C)** The half-life and **(D)** synthesis rate of SOD1 were determined from the Dendra2 signal in NSC-34 cells expressing soluble and insoluble SOD1^A4V^-Dendra2. Data represent mean ± SEM (*n* = 4 replicates, 59 single cell measurements, taken from data set in Figure 1). Statistical significance between groups was determined with an unpaired Student’s *t*-test (**p < 0.01). Scale bars: 10 μm.

We have previously shown that cells that contain inclusions have a smaller pool of free ubiquitin and an accumulation of a UPS reporter containing a CL1 degron peptide, suggesting that the UPS is overwhelmed (Farrawell et al., 2018, 2020). To investigate whether the aggregation of SOD1 impacts the rate of SOD1 degradation and SOD1 synthesis, we followed cells containing either predominately soluble or insoluble photoconverted SOD1^A4V^-Dendra2 and measured the degradation of SOD1-Dendra2-RFP signal (Figure 2A) and synthesis of SOD1-Dendra2-GFP signal (Figure 2B) in individual cells over time. We found that the turnover of SOD1^A4V^-Dendra2-RFP was slower in cells containing insoluble SOD1^A4V^ inclusions in comparison to cells containing only soluble SOD1^A4V^ (Figure 2A). The half-life of SOD1^A4V^ was significantly greater in cells containing SOD1^A4V^ aggregates (∼7 h) compared to cells expressing soluble SOD1^A4V^ (∼4 h) (Figure 2C). Interestingly, the synthesis of SOD1^A4V^-Dendra2-GFP in cells containing insoluble aggregates was slower than cells expressing soluble SOD1^A4V^ (Figure 2B), and although the difference was not significant, the rate of synthesis was decreased in cells containing insoluble SOD1^A4V^ inclusions (0.4 units/h) compared to cells expressing soluble SOD1^A4V^ (0.6 units/h) (Figure 2D).

### The Ubiquitin Proteasome System Slows Prior to Cu/Zn Superoxide Dismutase^A4V^ Aggregation

**FIGURE 3:**
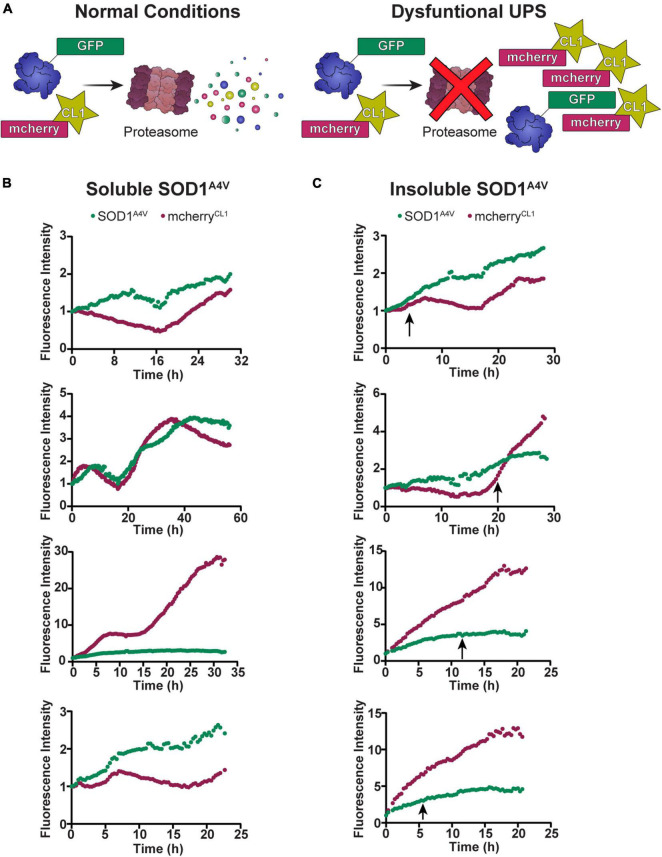
The UPS slows prior to SOD1^A4V^ aggregation. **(A)** Under normal conditions, mcherry^CL1^ is rapidly degraded by the proteasome, but if the UPS is overwhelmed or impaired, we see an accumulation of mcherry^CL1^ which can be measured by an increase in fluorescence intensity. The fluorescence intensity of SOD1^A4V^-GFP and mcherry^CL1^ was followed in NSC-34 cells expressing **(B)** soluble SOD1^A4V^-GFP and **(C)** insoluble SOD1^A4V^-GFP. Data represent individual cell traces with arrows indicating the time point at which insoluble SOD1^A4V^–GFP aggregates first appear.

Our previous work in NSC-34 cells showed that cells containing insoluble SOD1^A4V^ aggregates have impaired UPS function (Farrawell et al., 2018). Considering that the degradation of SOD1^A4V^-Dendra2-RFP was slowed in cells containing insoluble SOD1^A4V^ aggregates, we wondered whether the time at which the aggregation of SOD1^A4V^ occurs would correlate with changes in UPS function. To investigate this, we co-transfected NSC-34 cells with SOD1^A4V^-GFP and the fluorescent proteasome reporter mcherry^CL1^ (Figure 3A) and monitored changes in GFP and mcherry fluorescence in individual cells over time, paying particular attention to when insoluble SOD1^A4V^ inclusions developed. Overall, there was a trend for both SOD1^A4V^-GFP and mcherry^CL1^ fluorescence to increase over time in cells containing either soluble SOD1^A4V^ (Figure 3B) or insoluble SOD1^A4V^ (Figure 3C). However, we found no distinct pattern of mcherry^CL1^ fluorescence in relation to SOD1^A4V^-GFP aggregation (Figure 3C), suggesting that the UPS dysfunction that was observed with SOD1^A4V^-GFP expression happens prior to SOD1^A4V^ aggregation.

## Discussion

In line with previous studies, our results show that the turnover of mutant SOD1 occurs at a considerably faster rate than wild-type SOD1. Furthermore, we demonstrate that the aggregation of mutant SOD1^A4V^ impairs the turnover and synthesis of mutant SOD1 protein. These results conflict with previous reports that found no differences in SOD1 half-life (Farr et al., 2011), or levels of proteasome activity (Cheroni et al., 2005), regardless of the presence of aggregates in spinal cord tissue from G93A mice. However, given that motor neurons make up only 1–2% of neurons in the spinal cord (Chung et al., 1984), this discrepancy may stem from the analysis of the whole spinal cord, as opposed to focusing specifically on the motor neuron population.

It has been hypothesised that the accumulation of insoluble protein aggregates can overwhelm the UPS, activating mechanisms that lead to alterations in protein degradation and protein synthesis (Verhoef et al., 2002; Varanda et al., 2020). Our previous work has shown that the aggregation of ALS-associated proteins, including SOD1, leads to UPS dysfunction in NSC-34 cells (Farrawell et al., 2018, 2020). In addition, blocking the UPS with proteasome inhibitor has been shown to increase the level and prolong the half-life of mutant SOD1 proteins (Hoffman et al., 1996; Johnston et al., 2000). Taken together, these data are consistent with there being an intimate link between protein aggregation and UPS dysfunction. This prompted us to examine the temporal sequence of protein aggregation and the accumulation of the UPS reporter mCherry^CL1^. However, there was no apparent consistent correlation between UPS dysfunction and the time at which aggregation occurs, consistent with the idea that both degradation and synthesis slows in cells on the way to forming inclusions. It is likely then that protein aggregation and alterations to the rate of protein synthesis is a consequence of the level of misfolded SOD1 reaching a threshold that triggers a proteostasis collapse. Given the cell to cell variety we observed, the level of misfolded SOD1 that triggers proteostasis collapse may be cell-specific.

We show for the first time that the aggregation of SOD1 leads to the slowing of its synthesis. One possible explanation for the slowing of protein synthesis upon the aggregation of SOD1 is the activation of the Unfolded Protein Response (UPR). The UPR is a well-known response to protein misfolding and ER stress in ALS (Kikuchi et al., 2006; Saxena et al., 2009; Prell et al., 2012). Indeed, early stress-related responses have been detected in vulnerable motor neurons from fALS mice, followed by changes in the gene expression profile of UPR and UPS-related genes, which occur before the earliest denervation event in asymptomatic animals (Saxena et al., 2009). Our previous work also shows that supersaturated proteins are down-regulated in ALS (Yerbury et al., 2019), likely due to UPR induced changes.

The UPR uses three distinct pathways to sense disruptions to ER proteostasis and to trigger subsequent cellular responses (Walter and Ron, 2011). These stress responses depend on three master regulator proteins that all span the ER membrane enabling them to detect misfolded proteins [inositol-requiring enzyme-1 alpha (IRE1α), activating transcription factor 6 (ATF6), and PKR-like endoplasmic reticulum kinase (PERK)]. IRE1α and ATF6 both predominantly exert their effects via transcriptional reprogramming, while PERK exerts its effects on cellular translation through the phosphorylation of eukaryotic translation initiation factor 2α. Together these responses have the potential to target thousands of transcripts (Reich et al., 2020), slowing their overall synthesis rates. While SOD1 does not localize to the ER, the cytosolic misfolding and aggregation result in the dysfunction of the UPS which has been shown to trigger the UPR through the subsequent build-up of misfolded proteins in the ER due to restricted access to the proteasome.

Low levels of free ubiquitin, such as those we have observed in cells with SOD1 aggregates, result in a situation known as ubiquitin stress that may cause increased disassembly of ubiquitin conjugates (Flick and Kaiser, 2012). It is possible that the low level of free ubiquitin caused by the increase in misfolded and aggregated SOD1 provides a feedback loop that slows SOD1 degradation via the actions of deubiquitylating enzymes stripping ubiquitin in order to replenish the free ubiquitin pool. In addition, our previous results suggest that the proteins that are most supersaturated are likely to misfold under stressful conditions (Ciryam et al., 2017; Farrawell et al., 2018) further adding to the ubiquitin demand and overwhelming the system. Taken together, these data suggest that the slowing of protein synthesis and protein degradation are unlikely to be specific to SOD1 alone.

In conclusion, our data demonstrate that the aberrant accumulation of SOD1 aggregates in cells impairs the turnover of SOD1 protein, and highlight the importance of the protein degradation pathways, namely the UPS, in the progression of neurodegenerative diseases such as ALS. We believe that our data is consistent with a collapse in proteostasis from increased levels of misfolded SOD1 leading to aggregation, a slowing of protein degradation, and a slowing of protein synthesis. This is somewhat of a paradox given the apparent efficient turnover of mutant SOD1 found here and elsewhere. In future work, it will be important to understand the relationship between the apparent rapid and efficient degradation of mutant SOD1 and its accumulation in ALS.

## Data Availability Statement

The raw data supporting the conclusions of this article will be made available by the authors, without undue reservation.

## Author Contributions

JY conceived and supervised the study and provided new tools and reagents. JY and NF designed the experiments, analysed data, wrote the manuscript, and made the manuscript revisions. NF performed experiments. Both authors contributed to the article and approved the submitted version.

## Conflict of Interest

The authors declare that the research was conducted in the absence of any commercial or financial relationships that could be construed as a potential conflict of interest.

## Publisher’s Note

All claims expressed in this article are solely those of the authors and do not necessarily represent those of their affiliated organizations, or those of the publisher, the editors and the reviewers. Any product that may be evaluated in this article, or claim that may be made by its manufacturer, is not guaranteed or endorsed by the publisher.
